# Evaluation of the Influence of Genetic Variants of *SLC2A9* (GLUT9) and *SLC22A12* (URAT1) on the Development of Hyperuricemia and Gout

**DOI:** 10.3390/jcm9082510

**Published:** 2020-08-04

**Authors:** Katerina Pavelcova, Jana Bohata, Marketa Pavlikova, Eliska Bubenikova, Karel Pavelka, Blanka Stiburkova

**Affiliations:** 1 Department of Molecular Biology and Immunogenetics, Institute of Rheumatology, 128 50 Prague, Czech Republic; pavelcova@revma.cz (K.P.); bohata@revma.cz (J.B.); bubenikova@revma.cz (E.B.); pavelka@revma.cz (K.P.); 2 Department of Rheumatology, First Faculty of Medicine, Charles University, 128 50 Prague, Czech Republic; 3 Department of Probability and Mathematical Statistics, Faculty of Mathematics and Physics, Charles University, 186 75 Prague, Czech Republic; marketa@ucw.cz; 4 Department of Pediatrics and Adolescent Medicine, First Faculty of Medicine, Charles University and General University Hospital in Prague, 120 00 Prague, Czech Republic

**Keywords:** gout, hyperuricemia, urate transporters, sequencing, *SLC2A9*, *SLC22A12*

## Abstract

Urate transporters, which are located in the kidneys, significantly affect the level of uric acid in the body. We looked at genetic variants of genes encoding the major reabsorption proteins GLUT9 (*SLC2A9*) and URAT1 (*SLC22A12*) and their association with hyperuricemia and gout. In a cohort of 250 individuals with primary hyperuricemia and gout, we used direct sequencing to examine the *SLC22A12* and *SLC2A9* genes. Identified variants were evaluated in relation to clinical data, biochemical parameters, metabolic syndrome criteria, and our previous analysis of the major secretory urate transporter ABCG2. We detected seven nonsynonymous variants of *SLC2A9*. There were no nonsynonymous variants of *SLC22A12*. Eleven variants of *SLC2A9* and two variants of *SLC22A12* were significantly more common in our cohort than in the European population (*p* = 0), while variants p.V282I and c.1002+78A>G had a low frequency in our cohort (*p* = 0). Since the association between variants and the level of uric acid was not demonstrated, the influence of variants on the development of hyperuricemia and gout should be evaluated with caution. However, consistent with the findings of other studies, our data suggest that p.V282I and c.1002+78A>G (*SLC2A9*) reduce the risk of gout, while p.N82N (*SLC22A12*) increases the risk.

## 1. Introduction

Uric acid is the final product of purine metabolism in humans. If the balance between uric acid production and excretion is impaired, hyperuricemia can occur [1]. Since uric acid is poorly soluble, at higher concentrations, in the blood, monosodium urate crystals can form [2]. In the early stages, hyperuricemia is asymptomatic; however, over time, monosodium urate crystals can lead to gout, a form of inflammatory arthritis. In addition to gout, hyperuricemia is also associated with kidney disease, hypertension, cardiovascular disease, and type 2 diabetes mellitus [3,4,5].

Uric acid levels are influenced by various factors, such as the intake of dietary purines, the formation of endogenous purines, the excretion of uric acid via the kidneys and intestines, genetic predisposition, medications, and health conditions [1,6]. Different studies indicate that genetic factors are involved in 25–73% of cases [7]. GWAS studies have shown an association between hyperuricemia and gout and dysfunction of urate transporters [8,9]. These urate transport proteins are located primarily in the proximal tubules of the kidneys, and they are responsible for the excretion and reuptake of uric acid [1]. Variants of the genes that encode urate transporters are associated with both hyperuricemia and, in very rare cases, hypouricemia.

The major excretion urate transporter is ABCG2, while the GLUT9 and URAT1 proteins are important for reabsorption [6].

The *SLC2A9* gene (ENSG00000109667, solute carrier family 2 member 9, located on chromosome 4p16) encodes glucose transporter 9 (GLUT9). It occurs in two isoforms, GLUT9a, which is located on the basolateral membrane and GLUT9b, which is located on the apical membrane of the proximal tubules in the kidneys [2]. GLUT9 provides urate reuptake, and single-nucleotide polymorphisms (SNPs) of *SLC2A9* are associated not only with hyperuricemia and gout, but also with renal hypouricemia type 2 (OMIM(Online Mendelian Inheritance in Man) # 612076) [10].

The *SLC22A12* gene (ENSG00000197891, solute carrier family 22 member 12, located on chromosome 11q13) encodes urate transporter 1 (URAT1). Genetic variants of this gene lead, as in the case of *SLC2A9*, to hyperuricemia and gout and rare cases to hypouricemia type 1 (OMIM # 220150) [11].

The *ABCG2* gene (ENSG00000118777, located on chromosome 4q22) encodes the ATP-binding cassette sub-family G member 2 protein (ABCG2), which is the major secretor of uric acid. In addition to the kidneys, the ABCG2 protein is also located in the intestines, where it facilitates up to one-third of the excretion of uric acid [12]. In our previous work, we reported that genetic variants of the *ABCG2* gene (ENSG00000118777) increases the risk of developing gout, especially the common nonsynonymous variant p.Q141K (*rs2231142*) [13]. These variants are also associated with early disease onset, as confirmed by the findings of our study using a cohort of patients with pediatric-onset primary hyperuricemia and gout [14].

There are other urate transporters in the proximal tubules that are also responsible for uric acid transport, i.e., NPT1 (solute carrier family 17 member 1, *SLC17A1*), NPT4 (solute carrier family 17 member 3, *SLC17A3*), OAT4 (solute carrier family 22 member 11, *SLC22A11*), OAT10 (solute carrier family 22 member 13, *SLC22A13*), and MRP4 (ATP binding cassette subfamily C member 4, *ABCC4*) [2,15]. However, recent evidence suggests that these proteins have less impact on uric acid levels in the blood than GLUT9, URAT1, and ABCG2 [2,16].

The aims of our study were to identify which variants of the *SLC2A9* and *SLC22A12* genes existed in a cohort of 250 individuals with primary hyperuricemia and gout, and at what frequency they existed. We also intended to determine whether the variants were associated with uric acid levels and/or other important factors related to the development of hyperuricemia and gout. Polymorphisms of the *ABCG2* gene, biochemical parameters, and metabolic syndrome markers in this cohort were previously investigated in one of our other studies [13].

## 2. Experimental Section

The cohort consisted of 177 patients with primary gout and 73 patients with primary hyperuricemia under care from The Institute of Rheumatology. The gout diagnosis was determined using criteria developed by the American College of Rheumatology (ACR) Board of Directors and the European League Against Rheumatism (EULAR) Executive Committee [17]. The hyperuricemia group included individuals with elevated levels of uric acid (women > 360 µmol/L and men > 420 µmol/L). Increased levels of uric acid had to be repeatedly detected over a period of at least four weeks.

In our previous study, we examined 234 individuals from our cohort in search of pathogenic variants of the *ABCG2* gene [13]. The advantage of using this cohort was that we had already excluded individuals suspected of secondary hyperuricemia and secondary gout from our study. Using questionnaires filled out by physicians, we noted the presence of chronic kidney disease, active malignancy, diabetes, hypertension, or severe psoriasis. Furthermore, the age of onset of the first signs of gout and the patient’s family history of this disease were noted. In addition, an extensive biochemical examination was performed from peripheral blood samples. These same data were also recorded for an additional 16 individuals who were added to the cohort used in this, our current study.

Prior to data collection, all 250 participants signed informed consent. Ethics approval for this study was obtained from the Ethics Committee of the Institute of Rheumatology (reference number 6181/2015).

In order to identify SNPs of the *SLC2A9* and *SLC22A12* genes, PCR amplification and sequencing were performed. Peripheral blood was collected into EDTA tubes, and total DNA was isolated by using QIAamp DNA Mini Kits (Qiagen, Hilden, Germany) and stored immediately at −20 °C until analysis.

Specific PCR primers for coding regions of the *SLC2A9* and *SLC22A12* genes were designed, and PCR reaction conditions were optimized. For analysis of *SLC22A12*, the longest transcript, ENST00000377574, coding 553 amino acids and containing 10 exons was chosen. Other transcripts of *SLC22A12* were shorter but did not differ in the amino acid sequence. As for the *SLC2A9* gene, it occurs in two transcripts that differ in exon 3. The longer transcript, ENST00000264784, contains 540 amino acids and PCR primers were designed for all twelve exons. In the shorter transcript, ENST00000506583, coding 511 amino acids, exons 1 and 2 were missing. In addition to the twelve exons in *SLC2A9*, PCR primers were also designed for exon 3 in which the amino acid sequence differs, in exon 3, from the longer transcript, ENST00000264784. The remaining exons of the two transcripts have the same sequence.

PCR products were first verified using electrophoretic analysis with 2% agarose gels.

Following electrophoresis, Presto 96 Well PCR Cleanup Kits (Geneaid, New Taipei City, Taiwan) were used to purify PCR products.

To determine nucleic acid sequences, purified PCR products were analyzed using an Applied Biosystems 3130 Genetic Analyzer (Thermo Fisher Scientific, Waltham, MA, USA), i.e., a 4-capillary electrophoretic instrument based on the Sanger sequencing method.

For evaluation of the data, reference sequences of the *SLC2A9* and *SLC22A12* transcripts listed in the Ensembl database were needed. We used Lasergene (DNASTAR) software (version 10.1.2, www.dnastar.com) to search for SNPs having the sequences of the individuals in our cohort.

Data were summarized as medians with interquartile ranges (IQR) or as absolute and relative frequencies where appropriate. Continuous characteristics between patients with hyperuricemia and patients with gout were compared using the Wilcoxon two-sample test; categorical characteristics were compared using the Fisher exact test. The binomial test was used for comparisons of sample minor allele frequencies (MAF) with population MAFs; results with *p*-values < 0.0001 were considered statistically significant. Differences in MAF between patients with hyperuricemia and with gout were explored using the Fisher exact test. Associations of the allelic variants with biochemical measurements (serum uric acid, creatinine, FEUA) and anamnestic data (age of onset of hyperuricemia or gout) were explored using the Kruskal-Wallis nonparametric ANOVA.

Associations between the allelic variants and hypertension were examined using the Fisher exact test. The level of statistical significance was set at 0.05; the Benjamini-Hochberg adjustment for multiple comparisons was used wherever appropriate. All analyses were performed using statistical language and environment R, version 3.6.3 (www.r-project.org).

## 3. Results

The characteristics of the cohort are summarized in Table 1 and Table 2. Basic clinical data and biochemical data relevant for hyperuricemia are also included. The overview also indicates how many individuals have the p.Q141K variant of the *ABCG2* gene, which significantly increases the risk of gout since it reduces urate transport capacity.

An overview of the variations found in our cohort of 250 individuals of the *SLC2A9* and *SLC22A12* genes is presented in Table 3. No nonsynonymous variants were found of the *SLC22A12* gene; however, five synonymous variants were detected: p.N82N, p.H86H, p.H142H, p.A416H, and p.L437L. We also identified three intronic variants.

In the *SLC2A9* gene, we detected seven nonsynonymous variants. Six of them were found in transcript ENST00000264784 (p.G25R, p.T275M, p.D281H, p.V282I, p.R294H, p.P350L) and the p.A17T variant was detected in exon 3 of transcript ENST00000506583. We also identified five synonymous variants in transcript ENST00000264784: p.L108L, p.T125T, p.I168I, p.L189L, and p.S515S. In transcript ENST00000264784 of the *SLC2A9* gene, we detected 16 intron variants and a novel variant, c.1002 + 68C > T, which is not yet listed in the Ensembl (Ensembl Genome Browser, www.ensembl.org) and NCBI (National Center for Biotechnology Information, www.ncbi.nlm.nih.gov) databases. We analyzed this variant using the Human Splicing Finder; the result was that this mutation probably has no impact on splicing. By examining the intron-exon boundaries of exon 3 of transcript ENST00000506583, we discovered three additional intronic variants.

Statistical analysis using the binomial test revealed genetic variants that were significantly more common in our cohort of 250 individuals with hyperuricemia and gout compared to their frequency in the European population (data from the Ensembl database). The variants of the *SLC2A9* gene were p.L108L, p.T125T, p.L18L, c.151-60T>C, c.249+35C>T, c.249+119G>A, c.250-40A>G, c.410+49A>G, c.1002+72G>A, c.63+18delT, and c.-40-45G>A (*p* = 0). A higher allelic frequency was found in *SLC22A12* for variants c.662-7C>T and c.955-38G>A (*p* = 0). On the other hand, some variants of the *SLC2A9* gene had higher MAFs in the European population, namely p.V282I, p.A17T, c.535+67A>G, c.1002+78A>G, c.1113+9A>C, c.1114-89G>C, and c.-40-13T>C (*p* = 0).

Table 4 shows the results of the Fisher test comparing differences in the occurrence of genetic variants in individuals with hyperuricemia vs. patients with gout. Interestingly, variants p.A17T (OR (odds ratio) = 3.44, *p* = 0.0023, *p*-value adjusted = 0.0432) and c.-40-13T>C (OR = 3.18, *p* = 0.0306, *p*-value adjusted = 0.2510) of *SLC2A9* were observed to be more frequent in patients with gout. In contrast, variants c.249 + 119G > A (OR = 0.42, *p* = 0.0012, *p*-value adjusted = 0.0432), c.151-60T>C (OR = 0.49, *p* = 0.0035, *p*-value adjusted = 0.0432) and c.249+35C>T (OR = 0.49, *p* = 0.0042, *p*-value adjusted = 0.0432) were more frequently found in the hyperuricemia subgroup. All associations except for c.-40-13T>C were statistically significant after adjustment for multiple comparisons.

The results of the statistical evaluation of the associations between variants of the genes and serum uric acid levels and fractional excretion of uric acid are shown in Table 5. After adjustment for multiple comparisons, there were no statistically significant associations. We also evaluated the relationship between genetic variants and creatinine, hypertension, age of onset of hyperuricemia or gout, but no associations were detected.

Since we already knew the *ABCG2* gene sequencing results for the investigated cohort, we also focused on comparing the mutual occurrence of variants in the *ABCG2*, *SLC2A9,* and *SLC22A12* genes. As for the *ABCG2* gene, we focused on dysfunctional variants p.Q141K (*rs2231142*), p.R147W (*rs372192400*), p.T153M (*rs753759474*), p.F373C (*rs752626614*), p.T434M (*rs769734146*), p.S476P, and p.S572R (*rs200894058*) [13]. Concerning the *SLC2A9* and *SLC22A12* genes, we were particularly interested in nonsynonymous variants (p.G25R, p.T275M, p.D281H, p.V282I, p.R294H, p.P350L) and other variants known from the literature to be associated with hyperuricemia and gout, or vice versa, i.e., to reduce the risk of gout, namely p.N82N, p.H86H, p.H142H, p.L108L, p.I168I, c.1002+78A>G, and c.535+67A>G [18,19,20,21,22,23]. We found that individuals with any of the above-mentioned dysfunctional variants of *ABCG2* (except p.Q141K) were more likely to have the p.D281H allele in *SLC2A9* (*p* = 0.0389). An interesting finding was that individuals with any of the dysfunctional variants of *ABCG2* were less likely to have the homozygous variant p.P350L of *SLC2A9*. Furthermore, we found that individuals with the intronic variant c.1002+78A>G of *SLC2A9* were less likely to have dysfunctional variants of *ABCG2* (*p* = 0.014). Comparisons of the mutual occurrence of other variants did not show any statistically significant results, so only results for variants p.D281H, p.P350L, and c.1002+78A>G are summarized in Table 6, Table 7, Table 8 and Table 9.

## 4. Discussion

The main aims of our single center study were to (1) identify variants of the *SLC2A9* and *SLC22A12* genes, (2) determine their frequency compared to the European population, and (3) to evaluate the variants in relation to clinical, biochemical, and genetic data of a cohort with primary hyperuricemia and gout.

No nonsynonymous variants were found of the *SLC22A12* gene, which was highly conserved. This leads to an important question about the effect of synonymous and intronic variants on the development of hyperuricemia and gout. From variants detected in our cohort, we found references in the literature to three synonymous variants. In one study comparing the effect of single nucleotide polymorphisms on uric acid levels, the p.N82N variant was found to be associated with hyperuricemia [18]. Another synonymous variant, p.H86H, was also associated with hyperuricemia and gout [19,24,25]. In contrast, variant p.H142H reduces the risk of gout, according to authors of the study carried out on the Vietnamese population [20]. However, variants p.H86H and p.H142H are common in the European population as well as in our cohort. In contrast, the p.N82N variant rarely occurs; the MAF for the European population is 0.004; in our cohort, this variant occurred in two individuals.

In the *SLC2A9* gene, the variant p.V282I was found to be significantly more frequent in the European population (0.214) than in our cohort (0.118) (*p* = 0). According to a previously published study, this variant reduces the risk of gout [21]. Results regarding intronic variant c.1002+78A>G were also interesting. We found that this variant is significantly more common in the European population compared to our cohort. Our results also seem to be consistent with other research that found c.1002+78A>G reduces the risk of gout [23].

Functional studies have already been performed for all seven nonsynonymous variants that we found of the *SLC2A9* gene. Evaluation of urate uptake and expression was performed using *Xenopus laevis* oocytes. The results did not show significant differences (i.e., expression, location, and urate uptake) between native GLUT9 and proteins with nonsynonymous variants [26].

The association between genetic variants and serum uric acid levels and fractional excretion of uric acid cannot be interpreted with certainty. However, no association was found between creatinine, hypertension, age of onset of hyperuricemia or gout, and variants of the genes.

It is worth mentioning that in patients with primary gout, variants of the *ABCG2* gene occur more frequently than SNPs in *SLC2A9*, *SLC22A12,* and the other genes coding urate transporters. This matches our earlier observations, which showed that, in our cohort of 250 individuals with primary hyperuricemia and gout, the p.Q141K variant of *ABCG2* has a higher allele frequency relative to its allele frequency in the European population (0.24 vs. 0.09). Interestingly, the p.Q141K variant reduces urate transport capacity by up to 53% [16]. This variant also appears to be associated with a lower body mass index and C-reactive protein value [27].

Since different urate transporters are involved in the regulation of uric acid, it was interesting to compare the mutual occurrence of dysfunctional variants of the *ABCG2* gene with variants of the *SLC2A9* and *SLC22A12* genes. One study has already focused on the co-occurrence of selected variants of these genes, i.e., which variants p.H142H (*SLC22A12*), p.V282I (*SLC2A9*) or p.G141K (*ABCG2*) were associated with reduced uric acid excretion [28]. According to our results, variant p.D281H appears to occur more frequently along with the dysfunctional variants of the *ABCG2* gene, so this allele could contribute, together with other variants of *ABCG2*, to increased levels of uric acid. Results regarding two other variants, p.P350L and c.1002+78A>G, are also noteworthy, i.e., they occur more frequently in individuals who do not have dysfunctional variants of *ABCG2*. Taking into account that, according to the conclusion of another study, variant c.1002+78A>G reduces the risk of gout, our results suggest that c.1002+78A>G and p.P350L could reduce the risk of hyperuricemia and gout [23].

**Figure 1:**
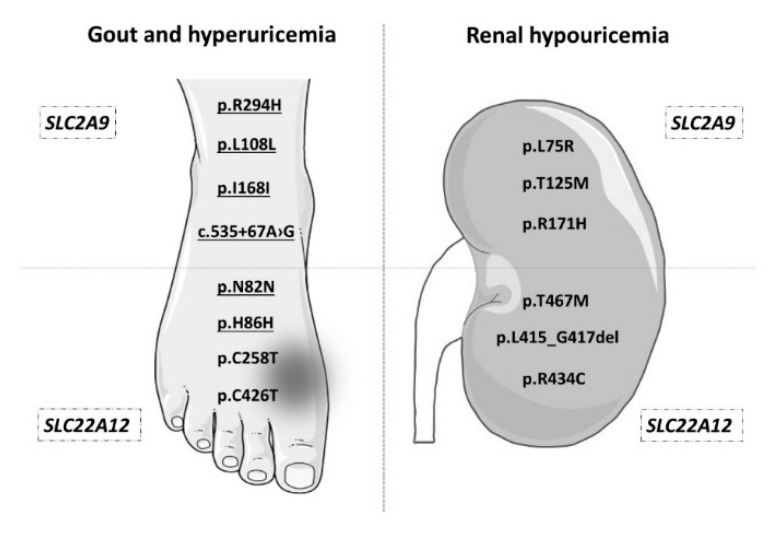
Genetic variants of *SLC2A9* and *SLC22A12* associated with hyperuricemia, gout, and renal hypouricemia. The picture shows some of the genetic variants that are, according to various studies, associated with elevated uric acid levels and increased risk of gout (on the left), or with rare renal hypouricemia (on the right). In the upper two quadrants, SNPs in the *SLC2A9* gene are shown, while in the lower quadrants, SNPs in *SLC22A12* are listed. The underlined genetic variants were found in our cohort. References to studies relating to genetic variants in this figure: p. R294H and p.I168I [22], p.L108L [31], c.535 + 67A > G [23], p.N82N [18], p.H86H [19], p.C258T and p.C426T [32], p.L75R [33], p.T125M [34], p.R171H [35], p.T467M and p.L415_G417del [29], p.R434M [36]. Foot and kidney images were copied from Servier Medical Art, by Servier (https://smart.servier.com; kidney image: https://smart.servier.com/smart_image/kidney-2/; foot image: https://smart.servier.com/smart_image/pied/) and adapted for the purposes of this article. Servier Medical Art by Servier is licensed under a Creative Commons Attribution 3.0 Unported License.

It is also important to mention that GLUT9 and URAT1 are referred to as proteins that are associated not only with hyperuricemia and gout, but also with hypouricemia since they are urate reuptake transporters. Figure 1 provides an overview of selected variants of the *SLC2A9* and *SLC22A12* genes that are associated with hyperuricemia and gout, or vice versa, with hypouricemia. None of the variants found in our cohort were associated with hypouricemia, which is not surprising in light of the characteristics of our cohort and also because renal hypouricemia is a very rare disease [29,30].

The lack of nonsynonymous variants of *SLC22A12* in our cohort was not so surprising since it is likely that these variants act as gout suppressors based on the reabsorption function of the URAT1 protein. This possibility is supported by a study that focused on nonsynonymous variant p.G774A, which is known to lead to the development of idiopathic renal hypouricemia in the Japanese population. In a cohort of 185 individuals with gout, the authors did not find p.G774A in any patient, while in healthy control subjects, it was present with a frequency of 2.3% [37]. Another study, which focused on two nonsynonymous variants p.R90H and p.W258X of *SLC22A12*, had very similar findings. These variants were also associated with renal hyperuricemia and were not detected in a large cohort of 1993 gout patients. In the group of healthy controls, these variants occur and reduce the risk of hyperuricemia [38]. However, the authors of another study came to different conclusions; they found nonsynonymous variants of the *SLC22A12* gene in 16 patients from a cohort of 69 individuals with gout. The p.C850G variant was detected in 11 patients from the cohort, while no nonsynonymous variants were found in the healthy controls. The unexpected results of this study can be explained by the different frequencies of the variants in diverse populations, i.e., the research was done in the Mexican population. Insight into this issue could provide useful information on the functional impact of the variants detected in this study, which is a question for further research [39].

The main advantage of our study was primarily its detailed genetic analysis of urate transporters GLUT9, URAT1, and the previously analyzed ABCG2 in a clinically and biochemically characterized cohorts of Czech patients with primary hyperuricemia and gout. However, our study has some limitations. A larger cohort would provide a clearer view of the effects of the variants of the *SLC22A12* and *SLC2A9* genes on the development of hyperuricemia and gout. This would also facilitate a more accurate statistical evaluation of less frequent variants in terms of uric acid levels. We also do not have data on the possible occurrence of asymptomatic urate crystal deposition in individuals with hyperuricemia, which could explain the association with genetic variants of the examined genes. It should also be noted that other urate transporters are involved in the transport of uric acid. Collectively these proteins act as a complex mechanism in the proximal kidney tubules, and it is very likely that the impaired function of one transporter could be compensated for by one or more of the other proteins.

However, more research on this topic needs to be done before the complexities of uric acid transport are fully understood, and other genes that encode urate transporters need to be examined.

## Figures and Tables

**Table 1:** Main demographic and genetic characteristics of the hyperuricemic (*n* = 68) and gout patients (*n* = 182).

	All (Number)	All (%)	Hyperuricemic (Number)	Hyperuricemic (%)	Gout (Number)	Gout (%)	Fisher Test *p*-Value
sex (men/women)	214/36	85.6/14.4	48/20	70.6/29.4	166/16	91.2/8.8	0.0002
familial occurrence of gout	97	59.8	31	48.3	66	63.5	0.0480
no treatment	58	23.2	30	44.1	28	15.4	<0.0001
treatment with allopurinol	175	70.0	38	55.9	137	75.3	
treatment with febuxostat	17	6.8	0	0.0	17	9.3	
p.Q141K-wild type	147	58.8	44	64.7	103	56.6	0.3682
p.Q141K-heterozygous variant	87	34.8	19	27.9	68	37.4	
p.Q141K-homozygous variant	16	6.4	5	7.4	11	6.0	
hypertension	100	52.8	24	58.6	76	50.6	0.3551

Fisher exact test for comparisons between categorical variables in hyperuricemia and gout cohorts. p.Q141K, variant of the *ABCG2* gene.

**Table 2:** Main clinical and biochemical characteristics of the hyperuricemic (*n* = 68) and gout patients (*n* = 182).

	All Median (IQR)	All Range	Hyperuricemic Median (IQR)	Hyperuricemic Range	Gout Median (IQR)	Gout Range	Wilcoxon Test *p*-Value
age of onset [years]	40.0 (28.0)	1.2–84	27.0 (40.5)	1.2–76	42.0 (24.0)	11–84	0.0026
age [years]	51.5 (25.0)	3–90	36.0 (42.0)	3–78	54.0 (21.0)	11–90	<0.0001
BMI	28.4 (5.8)	16–50	28.1 (6.4)	16–41	28.4 (5.4)	19.5–50	0.0822
WHR	1.0 (0.1)	0.6–1.7	1.0 (0.1)	0.7–1.3	1.0 (0.1)	0.6–1.7	0.0038
SUA off treatment [µmol/L]	460.0 (123.8)	181–683	446.0 (111.0)	253–608	462.0 (124.5)	181–683	0.6298
SUA on treatment [µmol/L]	375.0 (134.0)	163–808	424.0 (140.0)	240–628	372.0 (128.0)	163–808	0.0515
FEUA [fraction]	3.6 (1.7)	0.8–20	3.8 (2.0)	1.6–20	3.6 (1.6)	0.8–14.3	0.6066
GFR.MDRD	86.0 (27.6)	24–426	88.0 (36.0)	28–426	86.0 (26.0)	24–154	0.2312
serum creatinine [µmol/L]	80.5 (19.8)	26–226	79.0 (19.2)	26–132	81.5 (20.5)	47–226	0.0240
CRP	3.5 (6.4)	0.2–224.4	1.9 (4.6)	0.2–153.1	4.0 (6.4)	0.2–224.4	0.0025

Wilcoxon two-sample test for comparisons between continuous variables in hyperuricemic and gout cohorts. IQR, interquartile ranges; WHR, waist-hip ratio; SUA, serum uric acid; FEUA, excretion fraction of uric acid; GFR.MDRD, estimation of glomerular filtration rate; CRP, C-reactive protein. Note: These are data from the initial examination at the Institute of Rheumatology. At this time, uric acid levels were in the reference range in five individuals diagnosed with hyperuricemia.

**Table 3:** SNPs in *SLC2A9* and *SLC22A1*2 that were identified in a cohort of 250 patients with primary hyperuricemia and gout.

Variant	Gene	Region of the Gene	Reference SNP Number	Wild Type Homozygotes (Number)	Wild Type/Variant Heterozygotes (Number)	Variant Allele Homozygotes (Number)	Allelic Variant MAF	European Population MAF	Binomial Test *p*-Value
p.G25R, c.73G>A	*SLC2A9*	exon 1	*rs2276961*	44	109	97	0.606	0.528	0.0005
p.R294H, c.881G>A	*SLC2A9*	exon 7	*rs3733591*	161	78	11	0.200	0.191	0.6087
p.V282I, c.844G>A	*SLC2A9*	exon 7	*rs16890979*	195	51	4	0.118	0.214	0.0000
p.T275M, c.824C>T	*SLC2A9*	exon 7	*rs112404957*	244	6	0	0.012	0.009	0.4690
p.D281H, c.841G>C	*SLC2A9*	exon 7	*rs73225891*	238	12	0	0.024	0.029	0.5945
p.P350L, c.1049C>T	*SLC2A9*	exon 8	*rs2280205*	54	123	73	0.538	0.484	0.0176
p.A17T, c.49G>A	*SLC2A9*	exon 3	* rs6820230	222	0	28	0.112	0.297	0.0000
p.L108L, c.322T>C	*SLC2A9*	exon 3	*rs13113918*	7	48	195	0.876	0.800	0.0000
p.T125T, c.375G>A	*SLC2A9*	exon 3	*rs10939650*	10	58	182	0.844	0.752	0.0000
p.I168I, c.504C>T	*SLC2A9*	exon 4	*rs3733589*	237	13	0	0.026	0.045	0.0397
p.L189L, c.567T>C	*SLC2A9*	exon 6	*rs13125646*	7	47	196	0.878	0.801	0.0000
p.S515S, c.1545C>T	*SLC2A9*	exon 12	*rs144428359*	243	7	0	0.014	0.007	0.0944
c.150+24A>G	*SLC2A9*	intron 1–2	*rs2276962*	241	9	0	0.018	0.042	0.0050
c.150+65C>T	*SLC2A9*	intron 1–2	*rs2276963*	239	11	0	0.022	0.054	0.0007
c.151-60T>C	*SLC2A9*	intron 1–2	*rs2240722*	44	52	154	0.720	0.528	0.0000
c.249+35C>T	*SLC2A9*	intron 2–3	*rs2240721*	42	46	162	0.740	0.528	0.0000
c.249+119G>A	*SLC2A9*	intron 2–3	*rs2240720*	45	25	180	0.770	0.601	0.0000
c.250-40A>G	*SLC2A9*	intron 2–3	*rs28592748*	8	48	194	0.872	0.800	0.0000
c.410+29G>T	*SLC2A9*	intron 3–4	*rs16891971*	246	4	0	0.008	0.026	0.0069
c.410+49A>G	*SLC2A9*	intron 3–4	*rs772544951*	249	1	0	0.002	0.000	0.0000
c.535+67A>G	*SLC2A9*	intron 4–5	*rs3733590*	236	14	0	0.028	0.071	0.0000
c.681+25G>A	*SLC2A9*	intron 5–6	*rs13115193*	50	109	91	0.582	0.505	0.0006
c.681+13C>T	*SLC2A9*	intron 5–6	*rs202000076*	248	2	0	0.004	0.001	0.0901
c.682-31C>T	*SLC2A9*	intron 5–6	*rs4292327*	142	97	11	0.238	0.224	0.4528
c.1002+68C>T	*SLC2A9*	intron 7–8	*NA*	249	1	0	0.002	NA	NA
c.1002+72G>A	*SLC2A9*	intron 7–8	*rs1050991059*	249	1	0	0.002	0.000	0.0000
c.1002+78A>G	*SLC2A9*	intron 7–8	*rs6823877*	128	71	51	0.346	0.651	0.0000
c.1113+9A>C	*SLC2A9*	intron 8–9	*rs2280204*	196	48	6	0.120	0.200	0.0000
c.1114-89G>C	*SLC2A9*	intron 8–9	*rs114361719*	249	1	0	0.002	0.028	0.0000
c.63+18delT	*SLC2A9*	intron 3–4	* rs61256984	1	236	13	0.524	0.299	0.0000
c.-40-13T>C	*SLC2A9*	5’ UTR	* rs6449237	232	0	18	0.072	0.293	0.0000
c.-40-45G>A	*SLC2A9*	5’ UTR	* rs752032126	249	0	1	0.004	0.000	0.0000
p.N82N, c.246C>T	*SLC22A12*	exon 1	*rs3825017*	248	2	0	0.004	0.004	1.0000
p.H86H, c.258C>T	*SLC22A12*	exon 1	*rs3825016*	37	106	107	0.640	0.706	0.0014
p.H142H, c.426T>C	*SLC22A12*	exon 2	*rs11231825*	36	106	108	0.644	0.706	0.0027
p.A416A, c.1248A>G	*SLC22A12*	exon 7	*rs1630320*	0	0	250	1.000	1.000	1.0000
p.L437L, c.1309T>C	*SLC22A12*	exon 8	*rs7932775*	154	77	19	0.230	0.202	0.1191
c.662-7C>T	*SLC22A12*	intron 3–4	*rs373881060*	245	5	0	0.010	0.000	0.0000
c.1598+18C>T	*SLC22A12*	intron 9–10	*rs11231837*	152	79	19	0.234	0.199	0.0566
c.955-38G>A	*SLC22A12*	intron 5–6	*rs368284669*	248	2	0	0.004	0.000	0.0000

SNPs found in the *SCL2A9* gene in transcript ENST00000506583 are marked with an asterisk (*) sign, others come from longer transcript ENST00000264784. Genetic variants of the *SLC22A12* gene originate from transcript ENST00000377574. The minor allele frequency (MAF) in our cohort was compared to the European MAF using the binomial test.

**Table 4:** Comparison of genetic variants in individuals with primary hyperuricemia and patients with primary gout.

Variant	Individuals with Hyperuricemia				Patients with Gout				OR	Fisher Test *p*-Value	Benjamini-Hochberg Method: *p*-Value Adjusted
	Wild Type Homozygotes (Number)	Wild Type/Variant Heterozygotes (Number)	Variant Allele Homozygotes (Number)	Variant Allele MAF	Wild Type Homozygotes (Number)	Wild Type/Variant Heterozygotes (Number)	Variant Allele Homozygotes (Number)	Variant Allele MAF			
p.G25R	7	33	28	0.654	37	76	69	0.588	0.75	0.1829	0.7374
p.R294H	42	22	4	0.221	119	56	7	0.192	0.84	0.5300	1.0000
p.V282I	53	14	1	0.118	142	37	3	0.118	1.00	1.0000	1.0000
p.T275M	68	0	0	0.000	176	6	0	0.016	--	0.1966	0.7374
p.N281H	65	3	0	0.022	173	9	0	0.025	1.12	1.0000	1.0000
p.P350L	15	35	18	0.522	39	88	55	0.544	1.9	0.6875	1.0000
p.A17T	65	0	3	0.044	157	0	25	0.137	3.44	0.0023	0.0432
p.L108L	0	17	51	0.875	7	31	144	0.876	1.1	1.0000	1.0000
p.T125T	0	20	48	0.853	10	38	134	0.841	0.91	0.7834	1.0000
p.I168I	66	2	0	0.015	171	11	0	0.030	2.9	0.5291	1.0000
p.L189L	0	15	53	0.890	7	32	143	0.874	0.86	0.7589	1.0000
p.S515S	68	0	0	0.000	175	7	0	0.019	--	0.1978	0.7374
c.150+24A>G	66	2	0	0.015	175	7	0	0.019	1.31	1.0000	1.0000
c.150+65C>T	66	2	0	0.015	173	9	0	0.025	1.70	0.7351	1.0000
c.151-60T>C	4	17	47	0.816	40	35	107	0.684	0.49	0.0035	0.0432
c.249+35C>T	4	15	49	0.831	38	31	113	0.706	0.49	0.0042	0.0432
c.249+119G>A	4	10	54	0.868	41	15	126	0.734	0.42	0.0012	0.0432
c.250-40A>G	0	17	51	0.875	8	31	143	0.871	0.96	1.0000	1.0000
c.410+29G>T	67	1	0	0.007	179	3	0	0.008	1.12	1.0000	1.0000
c.410+49A>G	68	0	0	0.000	181	1	0	0.003	--	1.0000	1.0000
c.535+67A>G	65	3	0	0.022	171	11	0	0.030	1.38	0.7676	1.0000
c.681+25G>A	8	34	26	0.632	42	75	65	0.563	0.75	0.1855	0.7374
c.681+13C>T	68	0	0	0.000	180	2	0	0.005	--	1.0000	1.0000
c.682-31C>T	43	23	2	0.199	99	74	9	0.253	1.36	0.2383	0.8141
c.1002+68C>T	68	0	0	0.000	181	1	0	0.003	--	1.0000	1.0000
c.1002+72G>A	68	0	0	0.000	181	1	0	0.003	--	1.0000	1.0000
c.1002+78A>G	37	17	14	0.331	91	54	37	0.352	1.10	0.7514	1.0000
c.1113+9A>C	53	15	0	0.110	143	33	6	0.124	1.14	0.7585	1.0000
c.1114-89G>C	68	0	0	0.000	181	1	0	0.003	--	1.0000	1.0000
c.63+18delT	0	66	2	0.515	1	170	11	0.527	1.5	0.8407	1.0000
c.-40-13T>C	66	0	2	0.029	166	0	16	0.088	3.18	0.0306	0.2510
c.-40-45G>A	68	0	0	0.000	181	0	1	0.005	--	1.0000	1.0000
p.N82N	67	1	0	0.007	181	1	0	0.003	0.37	0.4704	1.0000
p.H86H	8	24	36	0.706	29	82	71	0.615	0.67	0.0749	0.4385
p.H142H	7	25	36	0.713	29	81	72	0.618	0.65	0.0586	0.4006
p.A416A	0	0	68	1.000	0	0	182	1.000	0.00	1.0000	1.0000
p.L437L	45	16	7	0.221	109	61	12	0.234	1.8	0.8119	1.0000
c.662-7C>T	67	1	0	0.007	178	4	0	0.011	1.50	1.0000	1.0000
c.1598+18C>T	44	17	7	0.228	108	62	12	0.236	1.5	0.9058	1.0000
c.955-38G>A	67	1	0	0.007	181	1	0	0.003	0.37	0.4704	1.0000

OR, odds ratio. In cases without a variant allele among hyperuricemic patients, the OR could not be enumerated (shown as a ‘–’ sign in the cell).

**Table 5:** The relationship between the detected variants and serum uric acid levels and fractional excretion of uric acid.

Variant	Median of Serum Uric Acid Levels [µmol/L]			Kruskal-Wallis ANOVA	Benjamini-Hochberg Method: *p*-Value Adjusted	Median of FEUA [%]			Kruskal-Wallis ANOVA	Benjamini-Hochberg Method: *p*-Value Adjusted
	Wild Type Homozygotes	Wild Type/Variant Heterozygotes	Variant Allele Homozygotes			Wild Type Homozygotes	Wild Type/Variant Heterozygotes	Variant Allele Homozygotes		
p.G25R	470	446	448	0.2114	0.653	3.6	3.5	3.7	0.5943	0.933
p.R294H	461	442	430	0.9211	0.921	3.6	3.6	4.2	0.2394	0.933
p.V282I	451	464	395	0.6131	0.735	3.7	3.3	3.1	0.2560	0.933
p.T275M	461	408	NA	0.1623	0.622	3.6	3.4	NA	0.9092	0.937
p.N281H	458	463	NA	0.4241	0.728	3.6	3.7	NA	0.6986	0.933
p.P350L	467	458	440	0.7566	0.830	3.6	3.6	3.7	0.9675	0.968
p.A17T	454	NA	476	0.3181	0.728	3.7	NA	3.6	0.5109	0.933
p.L108L	464	468	451	0.5179	0.734	3.3	3.6	3.6	0.5890	0.933
p.T125T	485	462	452	0.8198	0.867	3.6	3.6	3.6	0.6707	0.933
p.I168I	460	333	NA	0.1300	0.622	3.6	3.9	NA	0.5167	0.933
p.L189L	464	472	455	0.5865	0.735	3.3	3.2	3.7	0.3902	0.933
p.S515S	456	464	NA	0.6484	0.735	3.6	4.4	NA	0.5417	0.933
c.150+24A>G	460	312	NA	0.0215	0.622	3.6	3.9	NA	0.7131	0.933
c.150+65C>T	460	333	NA	0.1300	0.622	3.6	3.9	NA	0.4553	0.933
c.151-60T>C	473	444	446	0.0732	0.622	3.6	4.0	3.6	0.0656	0.736
c.249+35C>T	478	458	444	0.1310	0.622	3.6	4.0	3.6	0.0803	0.736
c.249+119G>A	482	458	444	0.0829	0.622	3.6	4.5	3.5	0.0037	0.127
c.250-40A>G	464	468	451	0.5179	0.734	3.2	3.6	3.6	0.5163	0.933
c.410+29G>T	460	568	NA	0.1830	0.622	3.6	4.2	NA	0.2784	0.933
c.410+49A>G	460	NA	NA	NA	NA	3.6	14.3	NA	NA	NA
c.535+67A>G	460	401	NA	0.3475	0.728	3.6	4.2	NA	0.1387	0.893
c.681+25G>A	477	451	442	0.0679	0.622	3.4	3.7	3.7	0.8218	0.933
c.681+13C>T	460	482	NA	0.6450	0.735	3.6	2.8	NA	0.1577	0.893
c.682-31C>T	442	462	482	0.2579	0.728	3.7	3.5	3.7	0.8507	0.933
c.1002+68C>T	460	600	NA	NA	NA	3.6	5.5	NA	NA	NA
c.1002+72G>A	460	437	NA	NA	NA	3.6	3.1	NA	NA	NA
c.1002+78A>G	450	451	469	0.3498	0.728	3.6	3.4	3.9	0.0865	0.736
c.1113+9A>C	462	441	385	0.6242	0.735	3.7	3.6	3.8	0.8498	0.933
c.1114-89G>C	460	NA	NA	NA	NA	3.6	2.9	NA	NA	NA
c.63+18delT	462	455	495	0.4499	0.728	3.1	3.6	3.8	0.6716	0.933
c.-40-13T>C	454	NA	477	0.1799	0.622	3.6	NA	3.6	0.9065	0.937
c.-40-45G>A	460	NA	548	NA	NA	3.6	NA	4.3	NA	NA
p.N82N	460	430	NA	0.8417	0.867	3.6	4.4	NA	0.3696	0.933
p.H86H	415	470	451	0.3873	0.728	3.6	3.6	3.7	0.4343	0.933
p.H142H	418	470	450	0.4299	0.728	3.6	3.6	3.7	0.4044	0.933
p.A416A	NA	NA	460	NA	NA	NA	NA	3.6	NA	NA
p.L437L	460	464	406	0.4105	0.728	3.6	3.6	3.4	0.7912	0.933
c.662-7C>T	460	414	NA	0.5885	0.735	3.6	2.7	NA	0.7552	0.933
c.1598+18C>T	460	468	406	0.3664	0.728	3.6	3.6	3.4	0.7872	0.933
c.955-38G>A	460	492	NA	0.4847	0.734	3.6	4.2	NA	0.4767	0.933

**Table 6:** Comparison of mutual occurrence of dysfunctional variants of *ABCG2* (p.R147W, p.T153M, p.F373C, p.T434M, p.S476P, and p.S572R) and the variant p.D281H in a cohort of individuals with hyperuricemia and gout.

		Without *ABCG2* Variants (Number)	Without *ABCG2* Variants (%)	Occurrence of Variants of *ABCG2* (Number)	Occurrence of Variants of *ABCG2* (%)	Total Number (without Distinction of Alleles in *ABCG2*)	Portion of the Whole Cohort (%)
p.D281H	wild type	233	95.9	5	71.4	238	95.2
	heterozygotes + homozygotes	10	4.1	2	28.6	12	4.8
total in the given column		243	100.0	7	100.0	250	100.0

Fisher’s Exact Test: *p*-value = 0.0389, odds ratio 9.11.

**Table 7:** Comparison of mutual occurrence of dysfunctional variants of *ABCG2* (p.R147W, p.T153M, p.F373C, p.T434M, p.S476P, and p.S572R) and the variant p.D281H in a cohort of individuals with gout.

		Without *ABCG2* Variants (Number)	Without *ABCG2* Variants (%)	Occurrence of Variants of *ABCG2* (Number)	Occurrence of Variants of *ABCG2* (%)	Total Number (without Distinction of Alleles of *ABCG2*)	Portion in the Whole Cohort (%)
p.D281H	wild type	169	96	4	66.7	173	95.1
	heterozygotes + homozygotes	7	4	2	33.3	9	4.9
total in the given column		176	100	6	100.0	182	100.0

Fisher’s Exact Test: *p*-value = 0.0295, odds ratio 11.6.

**Table 8:** Comparison of mutual occurrence of dysfunctional variants of *ABCG2* (p.Q141K, p.R147W, p.T153M, p.F373C, p.T434M, p.S476P, and p.S572R) and the variant p.350L in a cohort of individuals with hyperuricemia and gout.

		Without *ABCG2* Variants (Number)	Without *ABCG2* Variants (%)	Occurrence of Variants of *ABCG2* (Number)	Occurrence of Variants of *ABCG2* (%)	Total Number (without Distinction of Alleles of *ABCG2*)	Portion in the Whole Cohort (%)
p.P350L	wild type + heterozygotes	175	72	2	28.6	177	70.8
	homozygotes	68	28	5	71.4	73	29.2
total in the given column		243	100	7	100.0	250	100.0

Fisher’s Exact Test: *p*-value = 0.0239, odds ratio 6.38.

**Table 9:** Comparison of mutual occurrence of dysfunctional variants of *ABCG2* (p.Q141K, p.R147W, p.T153M, p.F373C, p.T434M, p.S476P, and p.S572R) and the variant c.1002+78A>G in a cohort of individuals with hyperuricemia and gout.

		Without *ABCG2* Variants (Number)	Without *ABCG2* Variants (%)	Occurrence of Heterozygous Variants of *ABCG2* (Number)	Occurrence of Heterozygous Variants of *ABCG2* (%)	Occurrence of Homozygous Variants of *ABCG2* (Number)	Occurrence of Homozygous Variants of *ABCG2* (%)	Total Number (without Distinction of Alleles of *ABCG2*)	Portion in the Whole Cohort (%)
c.1002+78A>G	wild type	68	47.6	48	54.5	12	63.2	128	51.2
	heterozygotes	52	36.4	17	19.3	2	10.5	71	28.4
	homozygotes	23	16.1	23	26.1	5	26.3	51	20.4
total in the given column		143	100.0	88	100.0	19	100.0	250	100.0

Fisher’s Exact Test: *p*-value = 0.014.
